# The Effect of Microcellular Structure on the Dynamic Mechanical Thermal Properties of High-Performance Nanocomposite Foams Made of Graphene Nanoplatelets-Filled Polysulfone

**DOI:** 10.3390/polym13030437

**Published:** 2021-01-29

**Authors:** Marcelo Antunes, Hooman Abbasi, José Ignacio Velasco

**Affiliations:** Department of Materials Science and Engineering, Poly2 Group, Technical University of Catalonia (UPC BarcelonaTech), ESEIAAT, C/Colom 11, 08222 Terrassa, Spain; hooman.abbasi@upc.edu (H.A.); jose.ignacio.velasco@upc.edu (J.I.V.)

**Keywords:** foams, polysulfone, graphene nanoplatelets, viscoelastic behavior, multifunctional

## Abstract

Polysulfone nanocomposite foams containing variable amounts of graphene nanoplatelets (0–10 wt%) were prepared by water vapor-induced phase separation (WVIPS) and supercritical CO_2_ (scCO_2_) dissolution. WVIPS foams with two ranges of relative densities were considered, namely, between 0.23 and 0.41 and between 0.34 and 0.46. Foams prepared by scCO_2_ dissolution (0.0–2.0 wt% GnP) were obtained with a relative density range between 0.35 and 0.45. Although the addition of GnP affected the cellular structure of all foams, they had a bigger influence in WVIPS foams. The storage modulus increased for all foams with increasing relative density and GnP’s concentration, except for WVIPS PSU-GnP foams, as they developed open/interconnected cellular structures during foaming. Comparatively, foams prepared by scCO_2_ dissolution showed higher specific storage moduli than similar WVIPS foams (same relative density and GnP content), explained by the microcellular structure of scCO_2_ foams. As a result of the plasticizing effect of CO_2_, PSU foams prepared by scCO_2_ showed lower glass transition temperatures than WVIPS foams, with the two series of these foams displaying decreasing values with incrementing the amount of GnP.

## 1. Introduction

Polysulfone (PSU) is an aromatic amorphous high-performance thermoplastic with high glass transition temperature and thermal-oxidative resistance, high strength and toughness, known for its resistance against hydrolysis and inherent fire resistance [1,2]. It is also resistant to acidic and basic attacks and can be processed at temperatures up to 400 °C due to its high melt thermal stability [3,4]. PSU foaming methods, such as CO_2_ dissolution [5,6,7,8,9,10], extrusion chemical foaming [11], and especially membrane formation [12,13,14,15,16,17], have been studied. Namely, given their hydrophobic and low surface energy, as well as relative easiness to form a controlled cellular structure by means of phase exchange, (micro)porous PSU has been used as anisotropic pore-flow membranes, also known as asymmetric porous membranes, for gas separation [18,19,20] (owing to the great permeability and O_2_/N_2_ selectivity of polysulfones [4]) and for high-performance reverse osmosis processes [21,22,23]. On the basis of its high thermal stability and thermal insulation, as well as obvious lightness, PSU foams have been considered in aircraft interior insulation components and have generated high interest in the aerospace, medical, and automotive sectors. However, the addition of carbon-based nanofillers into PSU is still at an incipient stage [4,24].

The high aspect ratio of nanofillers such as carbon nanotubes or graphene-based materials allows the preparation of high-performance multifunctional polymer-based nanocomposites [24,25,26]. Their addition into polymers can provide significant mechanical reinforcement at low nanofiller concentration [26,27], besides other functionalities such as thermal and/or electrical conduction, overcoming some of the technological barriers of polymers and enabling their use, for instance, as heat sinks [28] or in electronic packaging [29]. The mechanical performance of nanocomposites is highly dependent on the nanofillers chemical nature, content, geometry, and disposition/orientation, as well as on polymer type and the interactions established between nanofiller and polymer [26,27]. Previous studies of such materials have focused mainly on various types of carbon-based nanofillers, particularly, carbon nanotubes and graphene-based materials such as graphene nanoplatelets, graphene oxide, or reduced graphene oxide, owing to their intrinsically high mechanical properties. Among them, graphene nanoplatelets (GnP) have been considered in recent studies for enhancing the mechanical performance of polymer-based nanocomposites, focusing on the influence of nanocomposite’s preparation method, the dispersion and orientation of the nanoplatelets, and their aspect ratio and their interaction, in many cases, previously enhanced through nanofiller surface modification/functionalization, with the matrix. Results suggest certain constraints regarding the expected mechanical enhancement of the matrix through nanofiller addition, in some cases, even at relatively high nanofiller content, mainly related to insufficient dispersion and reagglomeration of nanofiller particles during processing.

The development of lightweight parts with enhanced specific properties and novel functionalities by combining foaming with the addition of nanosized carbon-based reinforcements to polymers has aroused a great interest in the last years [30]. Limited work has been done regarding the preparation and characterization of foams based on polysulfones with carbon-based nanofillers, with some examples of mechanical enhancement in nanocomposite membranes prepared by the WVIPS method [31,32,33,34]. As prior work, our research group has considered the preparation and characterization of polyetherimide foams reinforced with GnP and carbon nanotubes [35,36,37,38]. In terms of mechanical performance, the results indicate that the resulting foams displayed significantly higher specific storage moduli due to the combination of the high stiffness of the added graphene nanoplatelets, enhanced nanoplatelets’ distribution and dispersion throughout the matrix promoted by foaming, and finer cellular structure of the foams. With this idea in mind, in this work, the preparation of PSU-GnP nanocomposite foams by means of WVIPS and scCO_2_ dissolution and the analysis of their viscoelastic behavior is presented.

The originality of the present work is in the fact that for the first time, a comparative study is given between PSU-GnP foams prepared on the one hand by a well-known foaming method as is scCO_2_ dissolution, with the less common water vapor-induced phase separation, normally used for membrane preparation [39]. This second foaming method does not require melt-compounding of the material, as in the case of scCO_2_ dissolution foaming (necessity to melt-compound the nanocomposites in order to have the required foaming precursors for scCO_2_ dissolution), and enables to control in an easier way the density (also allowing to extend the final range of densities) and cellular structure of the resulting foams; and the study for the first time of the viscoelastic properties of such complex multiphase materials, critical in terms of defining the possibilities of the material for extended applications, analyzing how the foaming method, amount of GnP, and resulting relative density and cellular structure may affect the mechanical performance of the foams.

## 2. Materials and Methods

### 2.1. Materials

The following materials were used in this study: UDEL P-1700 polysulfone from Solvay (Brussels, Belgium), xGnP-Grade M15 graphene nanoplatelets from XG Sciences (Lansing, MI, USA), and *N*-methyl pyrrolidone (NMP) solvent from Panreac Química SA (Barcelona, Spain). For the analysis of the results presented in this work, UDEL P-1700 has a density of 1.24 g/cm^3^, a glass transition temperature of 185 °C and tensile and flexural moduli of 2480 and 2690 MPa, respectively, and the graphene nanoplatelets have a density of 2.20 g/cm^3^ (bulk density of the powder of 0.03–0.1 g/cm^3^, with an oxygen content <1%), thickness of 6–8 nm, average particle diameter of 15 μm, surface area of 120–150 m^2^/g, and a tensile modulus and strength parallel to the surface of 1000 and 5 MPa, respectively. More details about the materials are given in [40].

### 2.2. Foaming Methods

The following PSU-GnP nanocomposite foams were prepared for this study: two series of foams using the WVIPS method and a third series using scCO_2_ dissolution. In the case of foams prepared by the WVIPS method, GnP was initially dispersed in NMP (NMP-GnP solution) using a FB-705 ultrasonic processor (Fisher Scientific, Hampton, NH, USA) with a 12 mm solid tip probe applying a power of 95–130 W for 30 min at a controlled temperature of 50 °C. After ultrasonication, PSU pellets were dissolved in the NMP-GnP solution in order to obtain two different concentrations, i.e., 15 wt% PSU (15 PSU) or 25 wt% PSU (25 PSU), and stirred at 450 rpm during 24 h. Finally, 15 PSU and 25 PSU solutions were placed on a flat glass and foamed at room temperature by being exposed during 144 h to an air atmosphere with a controlled average humidity of 75%. PSU-GnP foams were prepared with these two PSU concentrations and the following amounts of GnP: 0, 1, 2, 5, and 10 wt%. It has to be stated that both PSU concentration (15 and 25 wt%) as well as range of GnP content (up till 10 wt%) are the result of prior testing and optimization. Above 10 wt% GnP, it was seen that NMP-GnP solutions started to have problems of proper GnP dispersion after ultrasonication, and especially, after PSU addition to these solutions, they had too high viscosity, leading later to problems of nonproper foaming. After complete foaming and stabilization, any possible NMP solvent remaining in the foams was removed by washing them in a 50/50 mixture of ethanol and water at 90 °C during 7 days, followed by vacuum drying at 100 °C for 7 additional days. Verification of complete NMP removal was done by confirming that, after the applied drying time, there was no weight loss associated to NMP removal (thermogravimetric analysis). More details about the complete foaming process can be found in [40].

PSU-GnP nanocomposite foams with variable amounts of GnP, namely, 0.0, 0.4, 0.7, 1.0, 1.5, and 2.0 wt% GnP, were prepared by scCO_2_ dissolution (PSU scCO_2_) by initially melt-mixing PSU with a 50 wt% ultrasonicated GnP-PSU masterbatch, produced following the same procedure as the NMP-GnP solutions prepared for WVIPS foams, using a Brabender Plasti-Corder 50EHT mixer (Brabender GmbH Co., Duisburg, Germany), molding the mixed PSU-GnP nanocomposites into circular-shaped disks (nominal thickness: 3 mm; diameter: 74 mm) using a hot-plate press IQAP LAP PL-15 (IQAP Masterbatch Group S.L., Barcelona, Spain) at 250 °C and 80 bar for 14 min and foaming said disks by dissolving scCO_2_ inside a high pressure vessel (CH-8610 Uster/Schweiz, Büchiglasuster, Switzerland) at a temperature of 185 °C and pressure of 180–210 bar for 5 h and applying a sudden pressure drop at a speed of around 0.3 MPa/s. The upper GnP content for foams prepared by scCO_2_ dissolution was set at a much lower value (2 wt% GnP), as above this concentration, the high amount of the platelet-like particles led to a high orientation of the nanoplatelets in the plane direction in the circular-shaped molded disks required to dissolve the scCO_2_, promoting CO_2_ loss and collapse of the cellular structure during foaming. More details about scCO_2_ dissolution can be found in [40].

### 2.3. Testing Procedure

Foam cellular structure was analyzed from micrographs obtained by scanning electron microscopy (SEM) using a JEOL JSM-5610 (Tokyo, Japan) microscope. SEM sample preparation included brittle fracture of the visualization surface using liquid nitrogen and later coating with a conductive layer of gold under inert atmosphere. Foam cell size (ϕ) was determined using a minimum of five 300× magnification micrographs [41]. The number of cells per volume of unfoamed materials (N_0_) was calculated according to:

(1) N0=(nA)32(ρcρf).

In this expression, n/A is the number of cells present in each micrograph per area of said micrograph, and ρ_c_/ρ_f_ is the volume expansion ratio, i.e., the reciprocal of relative density, ρ_c_ and ρ_f_ being the unfoamed and foamed nanocomposite densities, respectively. Both densities were measured using standard ISO-845, which considers the measurement of the apparent core density of cellular plastics, as compact skins formed during foaming in the case of foams prepared by scCO_2_ dissolution were removed prior to density measurement.

X-ray diffraction (XRD) was used to evaluate GnP’s dispersion/distribution by focusing on the analysis of the (002) diffraction plane of GnP. A PANalytical diffractometer (Almelo, The Netherlands), operating with CuKα (λ = 0.154 nm) at 40 kV and 40 mA and scanning from 2° to 60° with a scan step of 0.033°, was used.

The storage modulus (E′), loss modulus (E″), and tan δ of foams were measured as a function of temperature, and PSU’s glass transition temperature (T_g_) was determined as the maximum of both loss modulus and tan δ curves using dynamic-mechanical-thermal analysis (DMTA). A minimum of three rectangular-shaped samples, with a length of 35.5 ± 1.0 mm, a width of 12.5 ± 1.0 mm, and a thickness of 3.0 ± 0.5 mm, were analyzed per foam using a DMA Q800 equipment (TA Instruments, New Castle, DE, USA) in a single cantilever configuration heating from 30 to 250 °C at 2 °C/min and applying 0.02% and 1 Hz of dynamic strain and frequency, respectively. In the case of the foams obtained using scCO_2_ dissolution, compact skins generated during foaming were removed prior to the tests using sandpaper.

## 3. Results and Discussion

### 3.1. Cellular Structure

The relative density (ρ_f_/ρ_c_) of PSU-GnP nanocomposite foams prepared by WVIPS (15 PSU and 25 PSU) and scCO_2_ dissolution (PSU scCO_2_), as well as their composition and main cellular structure characterization results, are shown in Table 1.

**Figure 1:**
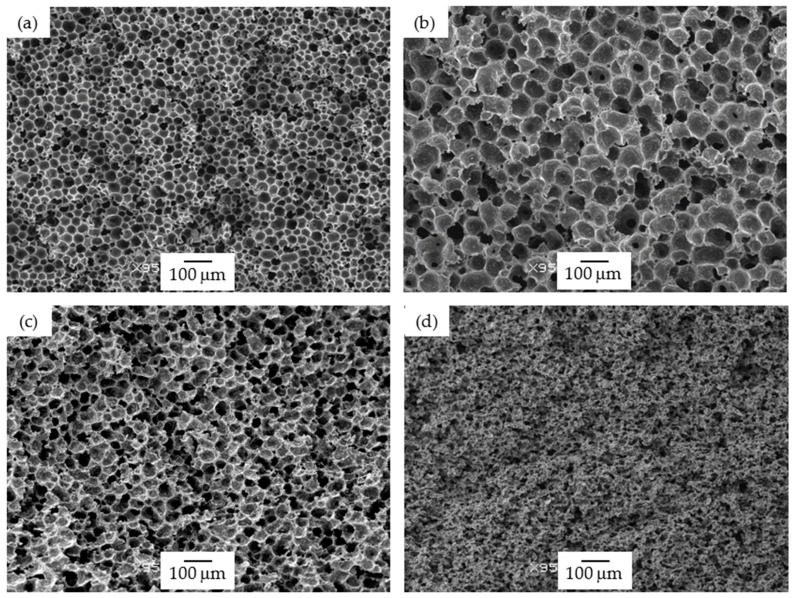
Micrographs at 95× magnification illustrating the cellular structure of 15 PSU foams: (**a**) 15 PSU; (**b**) 15 PSU-2 wt% graphene nanoplatelets (GnP), (**c**) 15 PSU-5 wt% GnP, and (**d**) 15 PSU-10 wt% GnP.

**Figure 2:**
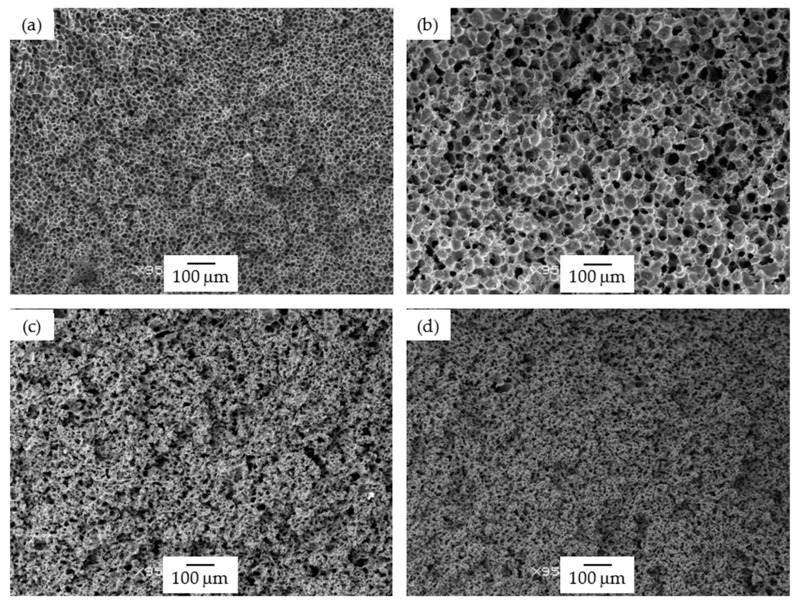
Micrographs at 95× magnification illustrating the cellular structure of 25 PSU foams: (**a**) 25 PSU, (**b**) 25 PSU-2 wt% GnP, (**c**) 25 PSU-5 wt% GnP, and (**d**) 25 PSU-10 wt% GnP.

Foams prepared using the WVIPS method (15 PSU and 25 PSU) showed a general increase in terms of cell size with adding GnP when compared to pure PSU foams, especially in the case of 15 PSU foams. The addition of GnP in foams prepared by WVIPS affected the kinetics of cell formation during NMP-water phase exchange due to the high affinity of GnP for NMP, inhibiting the exchange with water and globally slowing down the process. Interestingly, the addition of higher amounts of GnP, particularly, 10 wt% GnP in 15 PSU foams and 5 and 10 wt% GnP in 25 PSU foams, resulted in foams with open interconnected pores, once again the result of GnP’s affinity for NMP (see Figure 1d and Figure 2c,d).

**Figure 3:**
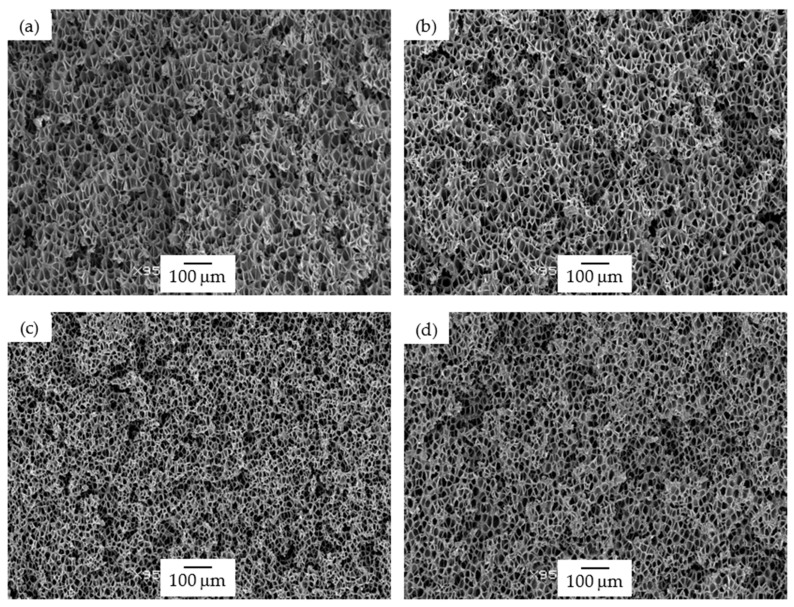
Micrographs at 95× magnification illustrating the cellular structure of PSU scCO_2_ foams: (**a**) PSU scCO_2_, (**b**) PSU scCO_2_-0.4 wt% GnP, (**c**) PSU scCO_2_-1.0 wt% GnP, and (**d**) PSU scCO_2_-2.0 wt% GnP.

Concerning foams prepared by scCO_2_ dissolution (PSU scCO_2_), first of all, they displayed relative densities comparable to 25 PSU foams and higher than those of 15 PSU foams; second, in terms of cellular structure, they showed cell sizes slightly smaller than 15 PSU and 25 PSU foams, with the addition of GnP resulting in a further slight decrease in the cell size when compared to pure PSU foams (see Figure 3). This could be explained by a barrier effect of graphene nanoplatelets to the diffusion of CO_2_. Increase in the concentration of GnP did not lead to further cell size reduction.

Cell density decreased for 15 PSU and 25 PSU foams with added GnP when compared to pure PSU foams. Nevertheless, no global tendency was observed with augmenting the amount of GnP. Similarly, foams prepared by scCO_2_ dissolution showed a general increase in cell density with adding GnP, with no clear trend being observed with augmenting the concentration of GnP.

### 3.2. Viscoelastic Behavior

**Figure 4:**
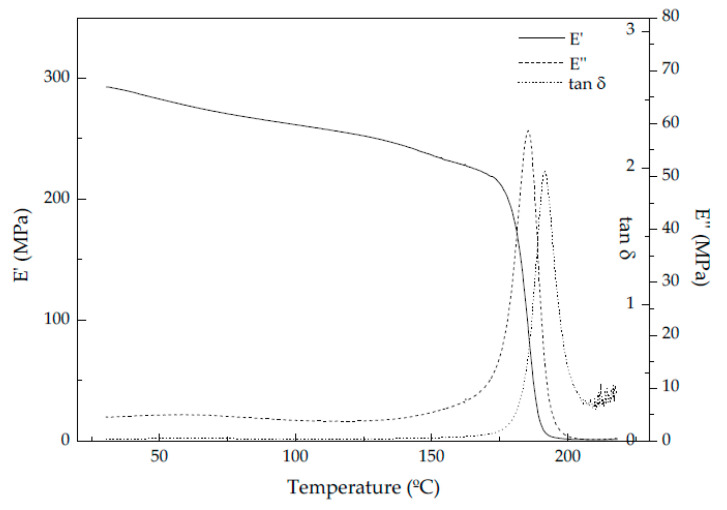
Typical storage modulus (E′), loss modulus (E″), and tan δ curves of PSU-GnP foams (15 PSU-2 wt% GnP).

**Figure 5:**
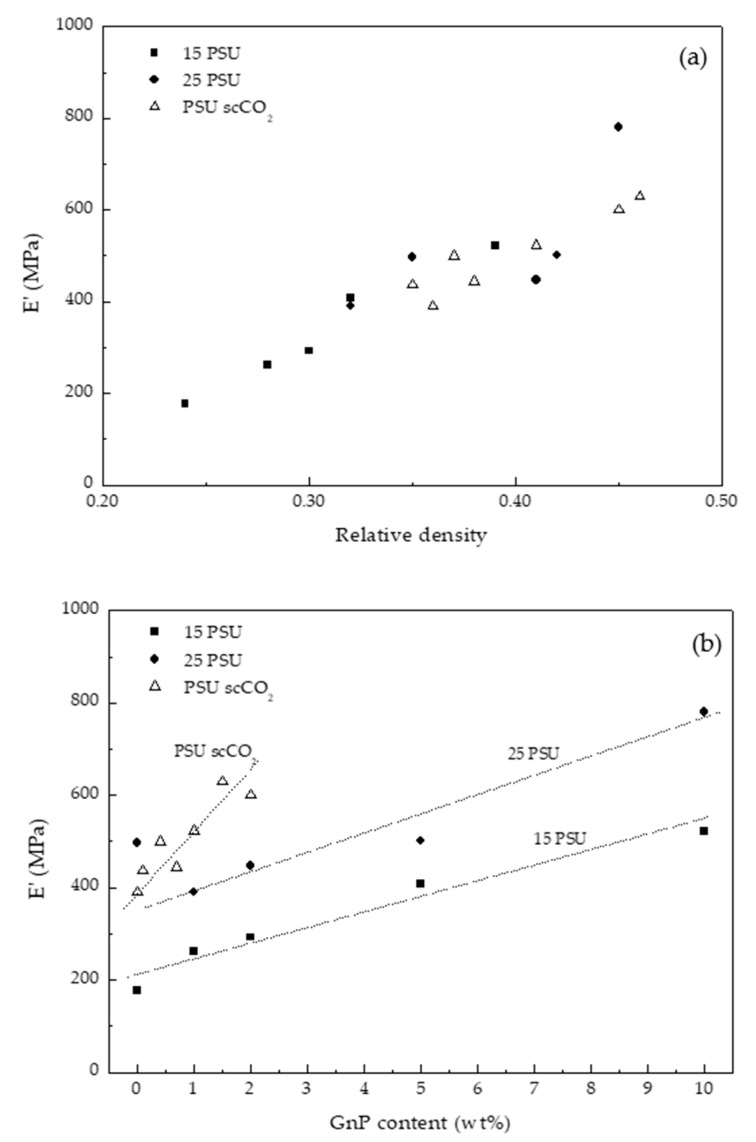
Storage modulus evolution of PSU and PSU-GnP nanocomposite foams with increasing (**a**) relative density and (**b**) GnP content.

The dynamic-mechanical-thermal characterization results, particularly, the storage modulus measured at 30 °C (E′), the specific storage modulus (E′_sp_, calculated by dividing E′ by the density of the foam), and the glass transition temperature (T_g_) of PSU measured at the maximum of the loss modulus (E″) and tan δ curves, of all PSU-GnP nanocomposite foams are presented in Table 2 and typical curves are represented in Figure 4 for 15 PSU-2 wt% GnP foams. The storage modulus is also represented for all PSU and PSU-GnP nanocomposite foams as a function of relative density and GnP content in Figure 5.

**Figure 6:**
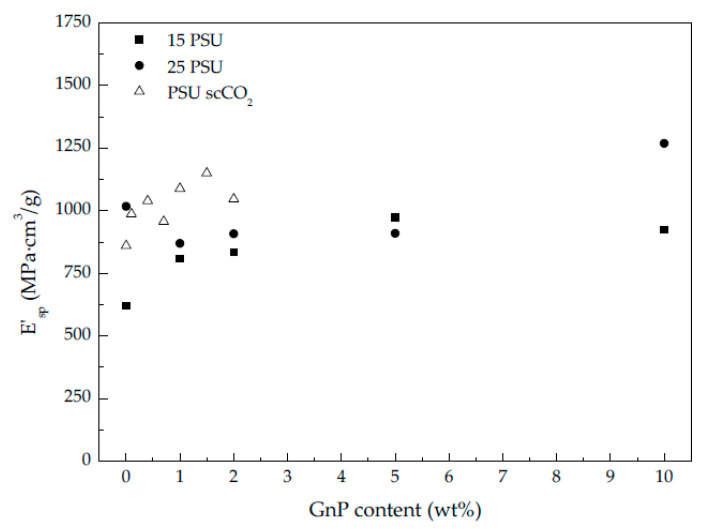
Specific storage modulus evolution of PSU and PSU-GnP nanocomposite foams with increasing GnP content.

As can be seen by the values presented in Table 2 and represented in Figure 5b and in Figure 6, the storage modulus and the specific storage modulus of 15 PSU and 25 PSU foams increased with incrementing the amount of GnP, except in the case of 15 PSU with 10 wt% GnP, which displayed a slightly lower value of the specific storage modulus than 15 PSU with 5 wt% GnP. This was related to the particular cellular structure formed by open interconnected pores of 15 PSU-10 wt% GnP foams when compared to the other 15 PSU foams, which displayed a closed-cell structure (see micrographs presented in Figure 1). This significantly different cellular structure could have been the result of GnP’s affinity for NMP, slowing down the kinetics of cell formation by hindering the phase exchange with water. In the same way, and although comparatively 25 PSU foams showed globally higher specific storage modulus values than their 15 PSU counterparts, related to the higher amount of PSU in 25 PSU foams and hence higher density, as well as smaller cell sizes, 25 PSU-5 wt% GnP foams displayed a lower specific storage modulus value when compared to 15 PSU-5 wt% GnP, as 25 PSU foams started to develop an open cell interconnected pore structure at 5 wt% GnP concentration (see Figure 2c).

**Figure 7:**
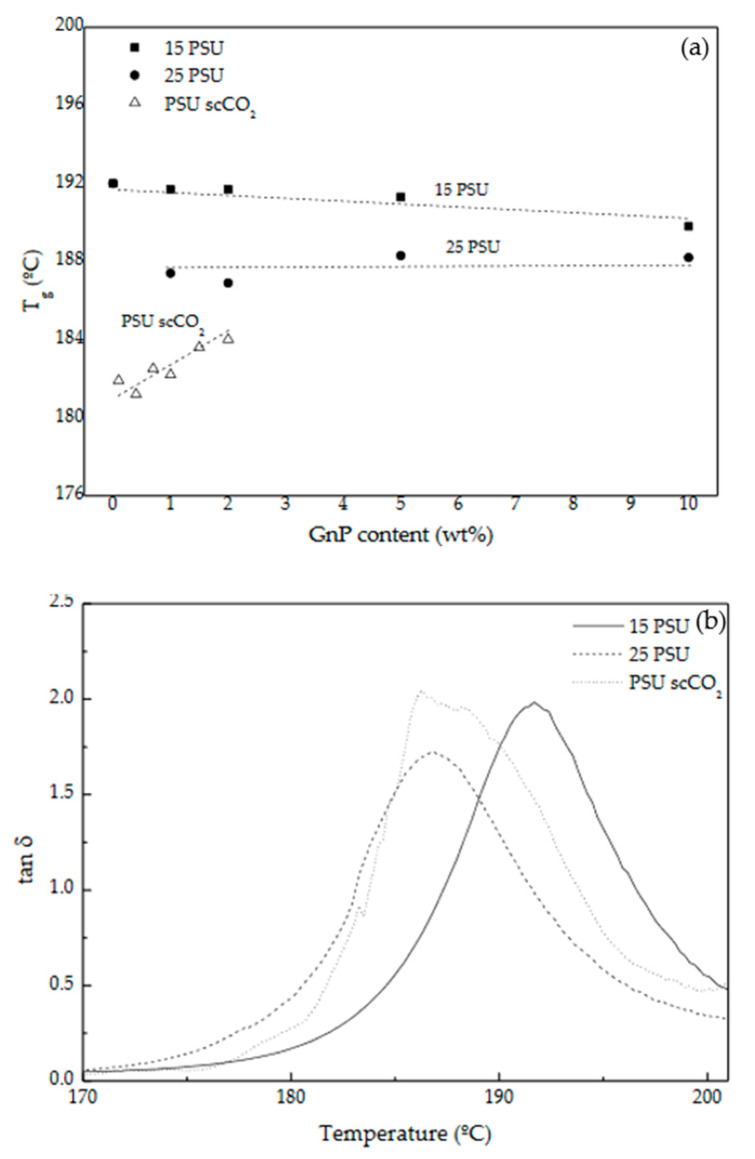
(**a**) Glass transition temperature of PSU measured at the maximum of tan δ for PSU and PSU-GnP nanocomposite foams with increasing GnP content and (**b**) comparative tan δ curves of 15 PSU, 25 PSU, and PSU scCO_2_ foams with 2 wt% GnP.

In terms of the evolution of the glass transition temperature (T_g_), which can be taken at the maximum of the loss modulus curves and related to the beginning of molecular mobility, and at the maximum of the tan δ curve (related to the end of the glass transition process), it globally decreased in both 15 PSU and 25 PSU foams with augmenting the amount of GnP when measured at the maximum of the loss modulus, especially in the case of 25 PSU foams, reaching T_g_ reductions >5 °C, and stayed almost unaltered when measured at the maximum of tan δ (Figure 7a). The addition of GnP promoted in both series of foams a plasticization effect, more significant in the case of 25 PSU foams, which was related to the interaction between PSU molecules and the highly flexible graphene nanoplatelets.

PSU-GnP foams prepared by scCO_2_ dissolution globally showed increasingly higher specific storage moduli with incrementing the amount of GnP (Figure 6), mainly related to the reinforcing effect of GnP, as all foams presented closed-cell structures with similar cell sizes (see micrographs presented in Figure 3). Only a slight reduction in E′_sp_ was observed at the highest added GnP amount (2 wt%), related to the slightly higher cell sizes of these foams. Comparatively, PSU scCO_2_ foams presented higher specific storage modulus values than 15 PSU and 25 PSU foams, even considering the lower concentrations of added GnP. When comparing foams having the same amount of GnP, for instance, PSU-2 wt% GnP foams prepared by scCO_2_ dissolution presented significantly higher E′_sp_ values than 15 PSU and 25 PSU foams with 2 wt% GnP (1046.5 MPa·cm^3^/g, compared to 833.6 and 907.7 MPa·cm^3^/g, respectively), which could be explained by the cellular structure of PSU scCO_2_ foams, more homogenous and with smaller cell sizes than 15 PSU and 25 PSU foams (compare the micrographs in Figure 1, Figure 2 and Figure 3 and the characterization results presented in Table 1).

Contrarily to 25 PSU foams prepared by WVIPS, the glass transition temperature of PSU scCO_2_ foams stayed almost unaltered with adding GnP when measured at the maximum of the loss modulus and increased slightly when measured at the maximum of tan δ (see Figure 7), as the important effect in its value was seen with considering scCO_2_ during foaming. So, when comparing foams obtained from both methods, those prepared by scCO_2_ dissolution showed much lower T_g_ values than PSU-GnP foams prepared by WVIPS. For instance, PSU-2 wt% GnP foams prepared by scCO_2_ dissolution displayed a T_g_ of 179.8 °C, almost 6 °C lower than 15 PSU-2 wt% GnP foams and 1.5 °C lower than 25 PSU-2 wt% GnP foams (see Figure 7b). This can be explained by the well-known plasticizing effect of scCO_2_ exerted on polymers, especially polymers containing benzene rings as is the case of PSU. Additional plasticization is expected when adding GnP, owing to the strong interaction between PSU’s benzene rings and GnP’s out-of-plane π bonds with CO_2_ molecules [42,43,44,45].

**Figure 8:**
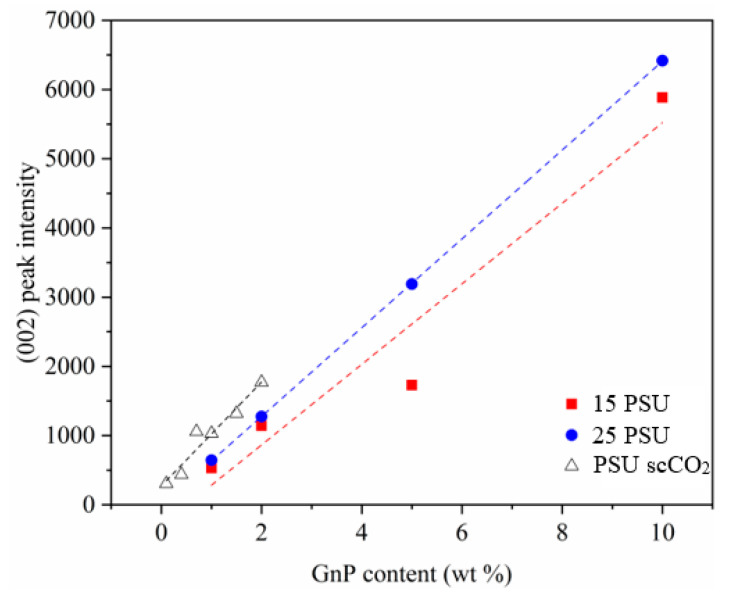
Normalized intensity value of the characteristic (002) diffraction plane of GnP for PSU and PSU–GnP nanocomposite foams and representation of the linear fits. The normalized peak intensity was determined by dividing the intensity by the density of each foam.

Although the comparatively lower intensity values of the characteristic (002) diffraction plane of GnP found at 2θ = 26.5° of WVIPS foams when compared to those prepared by scCO_2_ dissolution suggest a higher dispersion/distribution level of GnP (see values presented in Figure 8), which could be related to the lower viscosity of the mixing medium (NMP in the case of WVIPS foams) when compared to the viscosity of the melt during compounding (foams prepared by scCO_2_ dissolution), the noncomplete disappearance of this peak also shows that it was not possible to guarantee complete exfoliation and dispersion of individual graphene nanoplatelets. In any case, there was not a clear effect of the higher dispersion/distribution of GnP in WVIPS foams in enhancing the specific elastic modulus of foams, as scCO_2_ dissolution foams, with a comparatively lower degree of GnP dispersion/distribution, still displayed higher specific storage modulus values.

As a consequence, besides the influence of the developed cellular structure, we focused on the analysis of the effect of density on the final mechanical properties of foams, more specifically on their storage modulus. Although two-phase models are commonly used to predict the mechanical performance of polymer-based nanocomposites, they do not take into account the important effect of density on the final mechanical properties of foams. In the case of foams, the already shown increase in the storage modulus (E′_f_) by incrementing relative density (Figure 5) can be explained by the following power law expression, similar to the one suggested by Gibson and Ashby [40] for predicting the elastic modulus of cellular materials:

(2) $$\frac{{E^{\prime }}_{f}}{{E^{\prime }}_{c}}=C{\left(\frac{\rho_{f}}{\rho_{c}}\right)}^{n}.$$

In this expression, E′_c_ is the storage modulus of the reference unfoamed nanocomposite and C is a constant commonly assumed as being equal to 1 [46].

**Figure 9:**
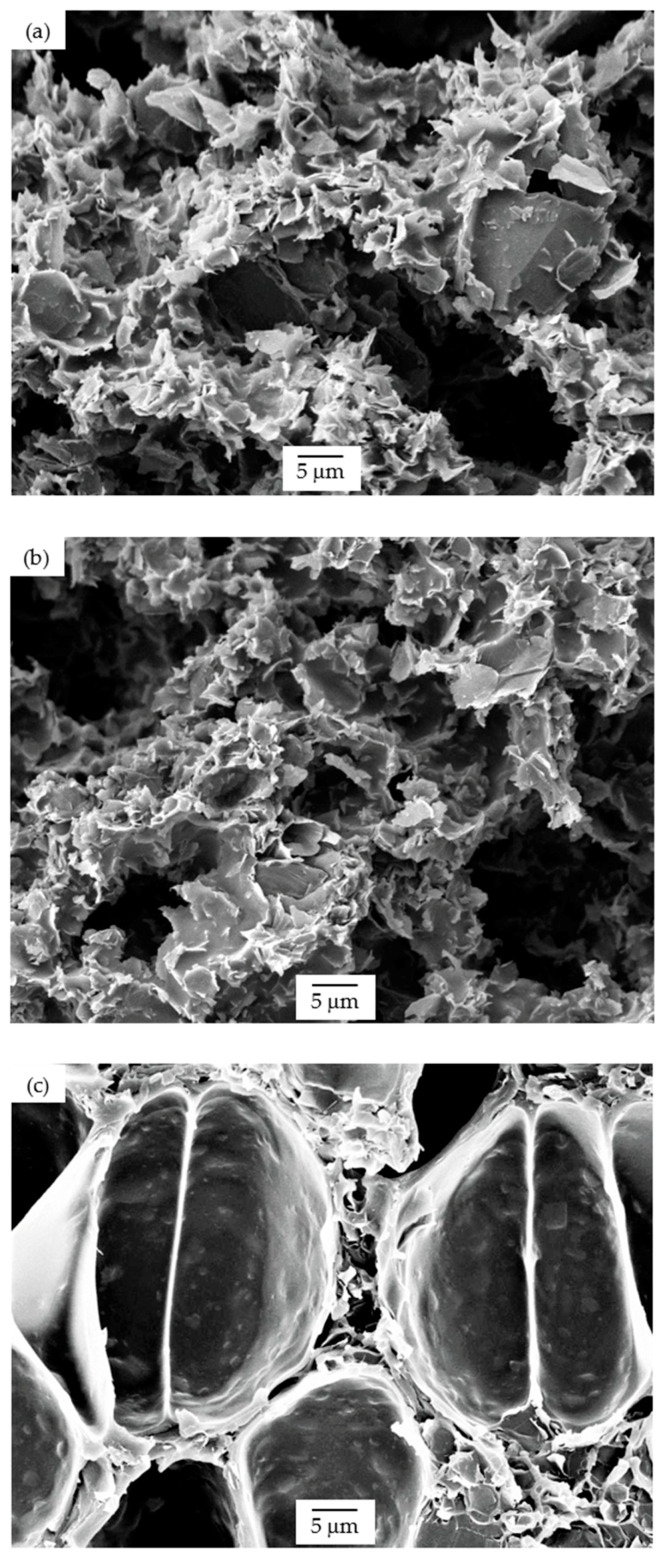
High-magnification micrographs illustrating the open interconnected pore cellular structure of (**a**) 15 PSU-10 wt% GnP and (**b**) 25 PSU-5 wt% GnP, and the characteristic closed-cell cellular structure of (**c**) PSU scCO_2_-2 wt% GnP.

Using an average value of the elastic modulus of all unfoamed nanocomposites determined from the representation of the storage modulus of all foams as a function of relative density (graphically represented in Figure 5), it was possible to calculate the value of the exponent n that appears in Equation (2) for each foam series. Comparatively, 25 PSU and PSU scCO_2_ foams showed n values much lower than 15 PSU foams (1.38 and 1.40, respectively for 25 PSU and PSU scCO_2_ foams and 2.29 for 15 PSU foams), indicating for these foams a global mechanical behavior closer to a characteristic closed-cell structure (see comparative high-magnification micrographs presented in Figure 9).

## 4. Conclusions

Regarding their cellular structure, PSU-GnP nanocomposite foams prepared by the WVIPS process displayed higher cell sizes and lower cell nucleation densities with incrementing the amount of GnP when compared to foams prepared by scCO_2_ dissolution. Particularly, those with lower GnP concentrations showed a closed-cell structure, while foams with a higher GnP content presented an open/interconnected structure. This suggests that the addition of GnP affected the kinetics of NMP-water phase exchange, slowing it down due to the affinity of GnP for NMP. Cell size slightly decreased by adding GnP for foams prepared by scCO_2_ dissolution, which could be the result of a barrier effect of graphene nanoplatelets to CO_2_’s diffusion during foaming.

The specific storage modulus of PSU-GnP foams increased with relative density and globally augmented with incrementing the amount of GnP, related to the reinforcing effect of GnP and to the globally closed-cell structure of the resulting foams. Nevertheless, among WVIPS foams, those with the highest amount of GnP that, as a result, developed a cellular structure formed by open interconnected pores, showed slightly lower specific storage moduli. On the contrary, even considering the lower concentrations of added GnP, PSU scCO_2_ foams presented higher specific storage moduli than foams prepared by WVIPS, especially in those cases where the same amount of GnP was considered, explained by the more homogeneous and finer cellular structure of scCO_2_ dissolution foams. In terms of the glass transition temperature, contrarily to 25 PSU foams prepared by WVIPS, whose values decreased with augmenting GnP concentration, PSU scCO_2_ foams displayed almost constant values independent of GnP amount. Comparatively, PSU scCO_2_ foams showed considerably lower T_g_ values than 15 PSU and 25 PSU foams, explained by the plasticizing effect of CO_2_.

This study may be seen as the starting point of the viscoelastic behavior analysis of PSU-GnP nanocomposite foams. Further testing based on multifrequency dynamic-mechanical testing would help to extend this analysis and elucidate the influence of the foaming process, presence and concentration of GnP, final relative density, and developed cellular structure on the viscoelastic characteristics of the foams, defining the possibilities of these lightweight materials for long-term applications.

## Figures and Tables

**Table 1:** Composition, relative densities, and cellular structure characterization results of polysulfone (PSU)-graphene nanoplatelets (GnP) nanocomposite foams.

Foam Series	Relative Density	GnP Content (wt%)	V_g_^1^	V_PSU_^2^	V_GnP_^3^	ϕ (μm)	N_0_ (cells/cm^3^)
15 PSU	0.24	0.0	0.759	0.241	0.000	29.7 ± 1.5	1.9 × 10^8^
	0.28	1.0	0.719	0.280	0.002	55.5 ± 1.7	3.3 × 10^7^
	0.30	2.0	0.705	0.292	0.003	56.7 ± 1.7	3.2 × 10^7^
	0.32	5.0	0.683	0.308	0.009	50.1 ± 1.5	5.1 × 10^7^
	0.39	10.0	0.615	0.362	0.023	Open cell	–
25 PSU	0.35	0.0	0.649	0.351	0.000	19.9 ± 1.0	5.2 × 10^8^
	0.32	1.0	0.677	0.321	0.002	21.2 ± 1.1	3.7 × 10^8^
	0.41	2.0	0.595	0.401	0.005	33.9 ± 1.4	1.0 × 10^8^
	0.42	5.0	0.580	0.408	0.012	Open cell	–
	0.45	10.0	0.547	0.426	0.027	Open cell	–
PSU scCO_2_	0.36	0.0	0.643	0.357	0.000	19.1 ± 0.6	2.1 × 10^8^
	0.35	0.1	0.655	0.345	0.000	17.3 ± 0.7	2.5 × 10^8^
	0.37	0.4	0.632	0.367	0.001	13.9 ± 0.6	3.4 × 10^8^
	0.38	0.7	0.620	0.378	0.002	15.1 ± 0.6	2.7 × 10^8^
	0.41	1.0	0.590	0.407	0.002	13.1 ± 0.5	4.1 × 10^8^
	0.46	1.5	0.539	0.457	0.004	13.8 ± 0.5	3.7 × 10^8^
	0.45	2.0	0.552	0.443	0.005	14.9 ± 0.6	2.1 × 10^8^

^1^ V_g_—volume fraction of gas. ^2^ V_PSU_—volume fraction of PSU. ^3^ V_GnP_—volume fraction of GnP.

**Table 2:** Dynamic-mechanical-thermal characterization results of PSU and PSU-GnP nanocomposite foams.

Foam Series	GnP Content (wt%)	Relative Density	E′ (MPa) ^1^	E′_sp_ (MPa·cm^3^/g) ^2^	T_g_ (°C) ^3^	
					E″	tan δ
15 PSU	0.0	0.24	176.5	621.5	186.4	192.0
	1.0	0.28	262.1	809.0	186.1	191.7
	2.0	0.30	292.6	833.6	185.6	191.7
	5.0	0.32	408.7	973.1	185.4	191.3
	10.0	0.39	522.6	923.3	184.9	189.8
25 PSU	0.0	0.35	497.4	1017.2	186.4	192.0
	1.0	0.32	389.7	869.9	181.1	187.4
	2.0	0.41	446.6	907.7	181.3	186.9
	5.0	0.42	501.3	909.8	183.4	188.3
	10.0	0.45	780.1	1268.5	181.2	188.2
PSU scCO_2_	0.0	0.36	389.7	860.3	179.4	183.8
	0.1	0.35	436.3	987.1	178.6	181.9
	0.4	0.37	499.1	1039.8	178.8	181.2
	0.7	0.38	443.3	957.5	179.7	182.5
	1.0	0.41	523.7	1088.8	179.2	182.2
	1.5	0.46	630.2	1150.0	179.1	183.6
	2.0	0.45	600.7	1046.5	179.8	184.0

^1^ Storage modulus measured at 30 °C. ^2^ Specific storage modulus at 30 °C (calculated by dividing E′ by the density of the foam). ^3^ Glass transition temperature at the maximum of the loss modulus (E″) and tan δ curves.

## Data Availability

The data presented in this study are available on request from the corresponding author.
